# The effect of gold kiwifruit consumed with an iron fortified breakfast cereal meal on iron status in women with low iron stores: A 16 week randomised controlled intervention study

**DOI:** 10.1186/1471-2458-10-36

**Published:** 2010-01-26

**Authors:** Kathryn Beck, Cathryn Conlon, Rozanne Kruger, Jane Coad, Welma Stonehouse

**Affiliations:** 1 Institute of Food, Nutrition and Human Health, Massey University, Auckland, New Zealand; 2 Institute of Food, Nutrition and Human Health, Massey University, Palmerston North, New Zealand

## Abstract

**Background:**

Dietary treatment is often recommended as the first line of treatment for women with mild iron deficiency. Although it is well established that ascorbic acid enhances iron absorption, it is less clear whether the consumption of ascorbic acid rich foods (such as kiwifruit) with meals fortified with iron improves iron status. The aim of this study is to investigate whether the consumption of ZESPRI^® ^GOLD kiwifruit (a fruit high in ascorbic acid and carotenoids) with an iron fortified breakfast cereal meal increases iron status in women with low iron stores.

**Methods/Design:**

Eighty nine healthy women aged 18-44 years with low iron stores (serum ferritin (SF) ≤ 25 μg/L, haemoglobin (Hb) ≥ 115 g/L) living in Auckland, New Zealand were randomised to receive an iron fortified breakfast cereal (16 mg iron per serve) and either two ZESPRI^® ^GOLD kiwifruit or a banana (low ascorbic acid and carotenoid content) to eat at breakfast time every day for 16 weeks. Iron status (SF, Hb, C-reactive protein (CRP) and soluble transferrin receptor (sTfR)), ascorbic acid and carotenoid status were measured at baseline and after 16 weeks. Anthropometric measures, dietary intake, physical activity and blood loss were measured before and after the 16 week intervention.

**Discussion:**

This randomised controlled intervention study will be the first study to investigate the effect of a dietary based intervention of an iron fortified breakfast cereal meal combined with an ascorbic acid and carotenoid rich fruit on improving iron status in women with low iron stores.

**Trial registration:**

ACTRN12608000360314

## Background

Iron deficiency is the most common nutritional deficiency worldwide and is common in premenopausal women [1]. In the 1997 New Zealand National Nutrition Survey low iron stores, iron deficiency and iron deficiency anaemia mainly affected women aged 15 to 44 years of age [2]. Iron deficiency is a concern due to its association with impaired work performance, cognitive function and immunity [3-5].

Mild iron deficiency can be effectively treated through dietary intervention [6]. This can include the addition of iron containing foods to the diet, such as foods fortified with iron [7], or improving the bioavailability of iron within meals [8]. Zimmerman et al [9] found that adding 12 mg of iron per day to snack foods increased iron status in Thai women with low iron stores. Snack foods fortified with ferrous sulphate improved iron status to a greater extent than snack foods containing electrolytic and hydrogen-reduced iron [9]. It is well established that ascorbic acid enhances iron absorption when added to meals [10,11]. Carotenoids including lutein and zeaxanthin have been shown to enhance iron absorption when added to a wheat based breakfast [12]. It is less clear whether the enhanced iron absorption by the addition of ascorbic acid or carotenoids to meals over time will improve iron status.

Studies investigating the effect of consuming ascorbic acid with meals have shown little or no effect on iron status [13-16]. Cook [13] found that consuming 1000 mg ascorbic acid with meals twice per day over 16 weeks did not improve iron status in seventeen healthy males and females who had a range of iron stores. Four subjects whose initial serum ferritin was <10 μg/L did however, improve their iron status. Subjects with normal iron stores are known to absorb iron less efficiently than women with depleted iron stores [17]. A study in eleven iron depleted women found that providing 500 mg ascorbic acid with three meals per day for five and a half weeks improved haemoglobin levels but not serum ferritin levels [15]. Using a cross over study design, Hunt et al [14] found that 500 mg ascorbic acid provided with meals three times per day for five weeks improved serum ferritin slightly but not significantly in twenty five iron depleted women consuming typical Western diets or diets of poor iron bioavailability. No changes in serum ferritin levels were seen in fourteen vegetarian subjects who consumed tofu alone or tofu and orange juice for 30 days in a cross over study [16]. Limitations of these studies include small sample sizes, three of the studies being of less than six weeks duration, and in some cases, the use of subjects with normal iron stores or whose iron levels was not reported. A well designed study by Garcia et al [18] found the addition of 25 mg of ascorbic acid as lime juice to two meals per day for eight months did not improve iron status in eighteen iron deficient Mexican women compared to a control group consuming a lime flavoured beverage with no ascorbic acid [18]. However, many of the reported studies have not stated the iron content of the meals to which ascorbic acid was added [13] or have only reported total daily iron intake [14,15,18]. In the one study that did report the iron content of the meals to which ascorbic acid was added, only 2.24 mg of iron was consumed at each meal [16]. Ascorbic acid promotes iron absorption by chelating iron in the intestinal lumen and promoting acidic conditions so that dietary iron is solubilised, preventing iron from binding to inhibitory ligands [19]. Ascorbic acid may therefore be more likely to improve iron status if consumed with meals containing substantial amounts of fortificant iron [20].

It is hypothesized that iron absorption from a breakfast cereal fortified with iron will be enhanced if consumed with kiwi fruit as kiwi fruit is a rich source of ascorbic acid (108.9 mg/100 g), lutein and zeaxanthin (307.9 μg/100 g) [21] and will consequently result in improved iron status. This study therefore aims to investigate whether consuming kiwifruit with a breakfast cereal fortified with 16 mg iron (1.92 mg natural iron, 14.08 mg iron in the form of ferrous sulphate) improves iron status in healthy women with low iron stores compared to a fruit low in ascorbic acid, lutein and zeaxanthin (banana).

## Methods/Design

**Figure 1:**
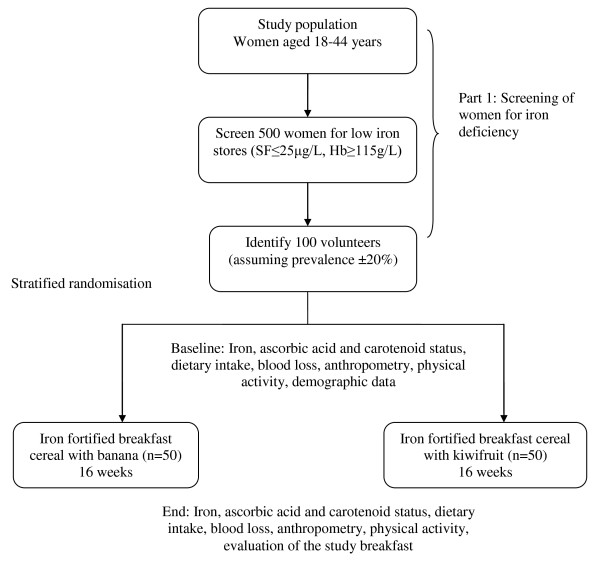
**Study design**. The effect of gold kiwifruit consumed with an iron fortified breakfast cereal meal on iron status in women with low iron stores: A 16 week randomised controlled intervention study

The study design is illustrated in Figure 1. The first phase of the study involved screening of 626 women to identify women with low iron stores. Phase two involved a randomised controlled intervention where women consumed iron fortified breakfast cereal and fruit every day at breakfast time for 16 weeks.

### Subjects

It was calculated that a minimum of 42 subjects with low iron stores would be required in each group to demonstrate a significant difference in serum ferritin of 2.5 μg/L at 80% power and 5% significance (two sided test). Power calculations were based on a standard deviation for serum ferritin of 4.1 μg/L (determined from a group of iron deficient women measured in our laboratory) (unpublished observation) and 2.5 μg/L was considered a possible clinically important difference. More subjects (n = 89) were recruited to allow for drop-outs from the study.

### Inclusion/exclusion criteria

Women aged 18-44 years with low iron stores (serum ferritin ≤ 25 μg/L and haemoglobin ≥ 115 g/L) were included in this study. A serum ferritin of ≤ 25 μg/L has been reported to be a clinically relevant cut off to demonstrate increased iron absorption with dietary factors [22]. Subjects were excluded from the study if they were pregnant, breastfeeding or if they had any known health problems likely to influence iron status including inflammatory bowel disease, coeliac disease, a history of gastric ulcers, disorders of red blood cells, menorrhagia, haemorrhoids, haematuria or malaria. Women who reported any allergies or intolerances to kiwifruit, bananas, milk or any of the components in the breakfast cereal were also excluded from the study. The women had to be willing to consume breakfast cereal, milk and kiwifruit or a banana for breakfast every day for a sixteen week period. Subjects had to be prepared not to donate blood nor to consume iron, ascorbic acid or calcium supplements (or supplements containing these nutrients) for the duration of the study, or to have regularly consumed iron supplements within the three months prior to participating in the study.

### Setting and recruitment

The study took place in Auckland, New Zealand. Subjects were recruited from the Auckland area using a variety of recruitment techniques. Women with low iron stores who had participated in a previous study [23] investigating the iron status of female university students conducted in our laboratory were invited to take part (n = 51). A web site was set up where women could find information about and register their interest in the study. Women were also recruited using newspaper advertisements that were placed in two local papers distributed free of charge to residents living on the North Shore of Auckland, New Zealand. An article written about the study was published in newspapers throughout Auckland region. A New Zealand magazine about healthy eating advertised the study via their monthly email newsletter. Forty six local primary schools were contacted and several advertised the study in their school newspaper. The study was also advertised using Facebook (a social networking site) and through emails sent to colleagues. The study was verbally promoted at local events attended predominantly by women (for example, a gym, a preschooler's library session, a cooking class and at a netball match). Posters and flyers were printed to advertise the study. A large number of flyers were delivered to houses on the North Shore, Auckland and researchers provided local businesses with flyers. Various businesses advertised the study on their websites.

### Screening

Women who expressed an interest in taking part in the study were provided with an information sheet and asked to complete a consent form and screening questionnaire and return these by post. The screening questionnaire asked questions to ensure subjects met the inclusion criteria for the study. A blood sample was taken at Diagnostic MedLab Ltd, Auckland or Massey University, Auckland by qualified phlebotomists. The blood sample was used to determine serum ferritin (SF), haemoglobin (Hb) from a complete blood count (CBC) and C-reactive protein (CRP). CRP was measured as raised CRP is associated with infection and may falsely elevate serum ferritin [24]. Women who had a serum ferritin ≤ 25 μg/L and haemoglobin ≥ 115 g/L were invited to continue with phase two of the study. Women with raised CRP (>5 mg/L) were invited to have a second blood test three weeks after the first blood test. All blood results were reviewed by a medical practitioner. Women with abnormal blood results including iron deficiency anaemia (SF<20 μg/L, Hb<115 g/L) were sent a copy of their results and advised to consult their general practitioner. Women with normal iron stores were sent a letter advising them of their results.

### The intervention - an overview

Refer to Figure 1. Women who met the criteria for the study were randomised into two groups. Both groups consumed a specially developed iron fortified breakfast cereal and milk at breakfast time for a 16 week period. One group consumed two ZESPRI^® ^GOLD kiwifruit with their breakfast. The other group (the control group) consumed a banana with their breakfast. Subjects were asked to consume this breakfast daily. Subjects came into the Human Nutrition Unit at Massey University before and after the 16 week period for baseline and final measures. At both visits the subjects gave a fasted blood sample, had anthropometric measures done, completed a face to face interview, and a physical activity questionnaire (see Table 1). Online questionnaires were completed at both visits. These included questionnaires about dietary intake and blood loss. Guidelines for completing the study were given at the first visit. Each visit took no longer than 2 hours.

**Table 1:** Outcome measures and testing methods used

Anthropometric and clinical assessments	
Height, weight, waist and hip circumference (baseline and end)	ISAK anthropometry protocol, electronic scales, stadiometer, Lufkin tape

**Questionnaires**	

Face to face interview (baseline)	Demographics, medical history, supplement use

Physical activity (baseline and end)	New Zealand Physical Activity Questionnaire Short Form (NZPAQ-SF) [26]

Dietary intake (baseline and end)	FFQ (iron containing foods, foods known to affect iron absorption)

Blood loss (baseline and end)	Adapted blood loss questionnaire [28]

Evaluation (end)	9 point hedonic scales to determine overall acceptability, appearance, flavour and texture [30]; 5 point just right scale regarding amounts of food [30]; food action rating scale (FACT) [31]; compliance diary

### Randomisation of subjects

Eighty nine women were recruited to take part in the intervention phase of the study. Subjects were matched in pairs according to their serum ferritin level and age. Each subject was randomly allocated to either the kiwifruit group or banana group by a coin toss. Forty four women were allocated to the kiwifruit group and forty five women to the banana group.

### Blood sampling and processing

A fasting blood sample was taken by a qualified phlebotomist, using a sterile Vacutainer Flashback needle and needle holder between 7.00 am and 10.00 am to avoid any diurnal variation, or the effects of recently eaten food on the indices measured. Subjects fasted (no food or beverages, excluding water) for 12 hours prior to this blood sample. Four millilitres of blood was collected into an EDTA tube and refrigerated at 4°C before being sent to Diagnostic MedLab for analysis of haemoglobin. Blood (3.5 mL) was collected into a SST tube for the analysis of serum ferritin and CRP and 10 ml of blood was collected into a red top tube for the analysis of soluble transferrin receptor and carotenoids. Both of these vacutainers were kept at ambient temperature for 30 minutes to allow time to clot, and then centrifuged for 10 minutes at 2500 g at 4°C within 2 hours. Six millilitres of blood was collected into an EDTA tube for the analysis of ascorbic acid. All tubes were protected from light post collection. The EDTA tube was centrifuged immediately at 2500 g at 4°C. This was separated without delay and 450 μL of the sample was mixed with 450 μL 10% trichloroacetic acid using a vortex mixer. Aliquots of serum were placed in 1.5 mL Nalgene System 100 cryogenic vials, immediately frozen on dry ice, protected from light and stored within 2 hours of collection at -80°C whilst awaiting analysis [25]. Samples collected at baseline and the end of the study for each subject were analysed in the same assay run to eliminate inter assay variability.

### Biochemical analysis

Details of methods and analysis are shown in Table 2.

**Table 2:** Biochemical analysis

Measure	Place of analysis	Method
**Blood analysis**		

Serum ferritin	Diagnostic Medlab, Auckland	Immunoturbidimetric test (Roche Diagnostics, Indianapolis) (Cat. No. 11661400)

Serum C-Reactive Protein	Diagnostic Medlab, Auckland	Particle enhanced immunoturbidimetric assay (Roche Diagnostics, Indianapolis) (Cat. No. 03002039)

Haemoglobin	Diagnostic Medlab, Auckland	SLS-Hb (sodium lauryl sulphate-Hb) method using automated haematology analyser XE-2100 (Sysmex Corporation, Auckland, NZ)

Soluble transferrin receptor	LabPlus, Auckland	Latex bound anti-sTfR antibodies react with antigen in sample to form antigen/antibody complex. Following agglutination measured turbidimetrically at 570 nm

Ascorbic acid	LabPlus, Auckland	Fe^3+ ^ions added to ascorbate which is oxidised to dehydroascorbate. Fe^2+ ^ions produced are measured by complexing them with ferrozine, the absorbance of the coloured complex is read at 560 nm. FeCl_3 _is added and incubation continued for 5 minutes. Concentration of ascorbate in specimen after suitable blank correction is proportional to change in absorbance over 5 minute period.

Carotenoids (Retinol, Alpha- & Gamma-Tocopherol; Lutein/Zeaxanthin, Lycopene, Alpha- & Beta-Carotene, Beta-Cryptoxanthin	Department of Medicine, University of Melbourne	HPLC method C18 column with absorbance detection at 292 nm for the tocopherols, 325 nm for retinol and 450 nm for carotenoids.

### Anthropometric measurements

Height, weight, waist and hip circumferences were measured in duplicate by a trained researcher using the International Society for the Advancement of Kinanthropometry (ISAK) protocol and standards. Quetelet's Body Mass Index (BMI) was calculated from height and weight.

### Questionnaires

At the first visit, subjects completed a face to face interview with a researcher regarding demographic and biographic information. This included questions on age, ethnicity; lifestyle questions (e.g. smoking) and eating patterns (e.g. vegetarian diets). A medical history was obtained from each subject which included questions regarding previous diagnosis or treatment for iron deficiency, blood loss, medication and supplement use in the past year.

At both visits, physical activity was measured using the validated New Zealand Physical Activity Questionnaire Short Form (NZPAQ-SF) [26]. Subjects were asked about the number of days and time spent brisk walking, or involved in moderate and vigorous physical activity over the past week.

Subjects completed two online questionnaires at baseline and end. The first questionnaire was a food frequency questionnaire (FFQ) which included iron containing foods and foods known to affect iron absorption. This was developed to determine overall dietary intake over the past month and to assess any changes in intake between groups over the course of the study. The FFQ was part of a computerised iron habits assessment tool (CIHAT) developed by the researchers [27] and is currently being assessed for its validity and reproducibility.

The second questionnaire asked about blood loss using a previously validated questionnaire [28]. This questionnaire was updated to include details on sanitary items (brand and absorbency) so that subjects could select the sanitary item that they used.

### The breakfast

Each breakfast consisted of 64 g breakfast cereal provided in a sealed individual packet, 150 ml milk and either a banana or two ZESPRI^® ^GOLD kiwifruit. The cereal was developed by food technologists at Hubbard Foods Ltd, Mangere, Auckland, NZ. Ferrous sulphate (Zenica BioPlus Pty Ltd, Australia) was added to a wheat flake cereal with apricot pieces to provide 16 mg iron per serve. Long Life Slim low-fat 0.1% ultra heat treated milk (Pams, New Zealand) was provided to eat with the cereal. ZESPRI^® ^GOLD kiwifruit stored at 1°C were provided by ZESPRI^® ^International Ltd. Bananas were sourced that were of similar size (approximately 100 g) and purchased in various stages of ripeness from market gardeners (MG Marketing, Auckland, NZ).

The breakfast cereal and milk were homogenised and analysed for their nutrient content at the end of the study (see Table 3). Ten packets of breakfast cereal and 3 one-L cartons of milk were sent to each venue from which a composite sample was obtained. Samples of kiwifruit and bananas were stored every two weeks at -80°C and protected from light. These were analysed at the end of the study. Each nutrition laboratory received bananas that were stored throughout the 16 week period. Two batches of kiwifruit were sent to each nutrition laboratory for analysis. The first batch consisted of three whole kiwifruit stored over the first six weeks of the study. The second batch consisted of three whole kiwifruit stored over the final six weeks of the study.

**Table 3:** Nutrient analysis

Nutrient	Place of analysis	Method of analysis
Gross energy (kJ)	Nutrition Lab, Massey University, Palmerston North	Bomb calorimetry

Moisture	Nutrition Lab, Massey University, Palmerston North	Moisture: Convection oven 105°C, AOAC 930.15, 925.10

Protein (g)	Nutrition Lab, Massey University, Palmerston North	Leco, total combustion method. AOAC 968.06

Ash	Nutrition Lab, Massey University, Palmerston North	Furnace 550°C, AOAC 942.05.

Fat - total (g)	Nutrition Lab, Massey University, Palmerston North	Fat: Acid hydrolysis/Mojonnier extraction. AOAC 954.02 (milk and cereal) Fat: Soxtec extraction, AOAC 991.36

Carbohydrate (total) (g)	Nutrition Lab, Massey University, Palmerston North	Calculation by difference

Dietary fibre (g)	Nutrition Lab, Massey University, Palmerston North	Enzymatic-gravimetric method. AOAC 991.43

Iron (mg), Calcium (mg), Copper (mg), Phosphorus (mg) & Zinc (mg)	Asure Quality, Auckland	Plasma emission spectrometry

Ascorbic acid (mg)	Nutrition Lab, Massey University, Palmerston North	HPLC

Vitamin A (as retinol) (μg)	Nutrition Lab, Massey University, Palmerston North	As Retinol, HPLC, AOAC 974.29 (4)

Lutein + zeaxanthin (μg), Beta-carotene (μg), Alpha-carotene (μg)	Plant and Food Research, Auckland	HPLC

Citric acid (mg)	Asure Quality, Auckland	Boehringerr Mannheim test kit (via titration)

Polyphenols (mg)	Plant and Food Research, Auckland	HPLC (sum of all peaks detected at 280 nm) with identification of specific compounds when possible

Phytic acid (mg)	Nutrition Lab, Massey University, Palmerston North	Megazyme kit, Megazyme International Ireland Ltd

Food samples (twenty random samples per product) were weighed for each product. Samples of cereal were taken from 4 separate cartons, banana from 1 weeks supply and kiwifruit across four separate deliveries. The mean (SD) weight of the cereal was 64.4 (3.7)g, peeled bananas weighed 104.1 (14.3)g, and one peeled kiwifruit weighed 85.4 (4.3)g.

Food composition data from ZESPRI^® ^International Ltd indicate that two ZESPRI^® ^GOLD kiwifruit (170.8 g) provided 186.0 mg ascorbic acid, 525.9 μg lutein and zeaxanthin, 6.0 μg of alpha carotene and 0 μg of beta carotene [21]. One banana (104.1 g) provided 9.1 mg ascorbic acid, 22.9 μg lutein and zeaxanthin, 26.0 μg of alpha carotene and 27.1 μg of beta carotene [29].

The breakfast cereal and milk were provided at the first visit and this was used as an opportunity to educate the subjects about the breakfast. The nutritional composition of the breakfast was explained. Subjects were asked to shake their packet of cereal before opening to ensure the iron was evenly mixed, and instructed to add 150 ml of milk to their cereal using the measuring jug provided. They were asked to eat the banana and kiwifruit with, immediately before or immediately after consuming the breakfast cereal. They were asked not to consume any other food or fluids (apart from tap water) with or one hour either side of consuming the breakfast. Instructions were given for eating the banana and the kiwifruit. Subjects were asked to discard the skin of the kiwifruit and to eat as much of the flesh as possible. It was emphasised that it was important to eat all of the cereal, milk, and fruit. The reasons for the instructions were individually explained to each subject, and also explained in a booklet that each subject was given to take home.

Subjects were provided with 120 packets of cereal and 17 L of milk to take home at their initial visit. All subjects were provided with a measuring jug to measure 150 ml of milk, as well as sufficient kiwifruit and bananas to last until the next delivery. Bananas were packaged and delivered weekly to subjects and kiwifruit were delivered every 2 weeks to subjects by a courier company. Subjects were provided with guidelines for storing the cereal, milk and fruit. It was recommended that the bananas be stored at room temperature and the kiwifruit in the refrigerator. Common scenarios that could potentially arise during the 16 week intervention were explained. For example, if subjects missed breakfast it was recommended that breakfast be consumed later in the day and the time eaten recorded in the diary.

### Compliance diaries

Subjects were asked to maintain their normal daily routine, for example, eating patterns (apart from the breakfast), physical activity, and alcohol consumption or smoking habits for the duration of the study.

All subjects were asked to complete a compliance diary. They were asked to tick if they ate breakfast each day, and record the time the breakfast was eaten. They were asked to comment on any food not consumed including amounts, or any other food eaten with the breakfast or consumed within 1 hour either side of the breakfast.

On a weekly basis subjects were asked questions regarding illness, medication use, symptoms experienced (including abdominal discomfort, bloating, constipation, diarrhoea, nausea, vomiting, headache and fatigue), changes to their normal daily routine, supplement use, and whether they were experiencing any practical problems with eating the breakfast.

Subjects were telephoned at week 2, 4, 8 and 12 of the study to provide support and answer any questions. Two emails were sent to subjects over the course of the study to maintain subject motivation. It was emphasised to subjects to contact the researcher (by phone or email) if they experienced any problems during the 16 week intervention.

At the final appointment, subjects were asked to confirm whether there were any major changes to their lifestyle or health while undertaking the study, including questions on blood donation and supplement use.

### Subject evaluation of the breakfast

At their final appointment all subjects completed an evaluation questionnaire. Nine point hedonic scales (ranging from dislike extremely to like extremely) were used to determine the overall acceptability, appearance, flavour and texture of the breakfast cereal and fruit, and the overall acceptability of the milk [30]. A five point just right scale was used to determine whether the amount of cereal, fruit and milk provided were acceptable. Statements on the scale ranged from "too little" to "just right" to "too much" [30]. Subjects completed a food action rating scale (FACT) [31] asking whether they would eat the study breakfast in the future. This scale ranged from "I would eat this food every opportunity I had" through to "I would eat this only if I was forced to". Subjects were asked to comment on any problems they experienced with consuming the breakfast, the time taken to get used to consuming the study breakfast; any side effects experienced while completing the study (positive or negative), the impact of the breakfast on daily food intake and their intentions regarding future breakfast consumption. Subjects were also asked to comment on researcher support and whether they would take part in a similar study again.

### Data handling and statistical analysis

Statistical analysis will be performed using SPSS for Windows Version 16 (SPSS Inc., Chicago, IL, USA) [32]. Name and contact details of all subjects are stored in a database, in individual files and in Microsoft Excel. Data will be entered in to a single Microsoft Excel spreadsheet with subjects identified only by their unique study number. All entered data will be checked by another member of the research team to ensure it has been entered accurately prior to statistical analysis.

Descriptive statistics will be used to describe the baseline population using mean (standard deviation), median (25, 75 percentile) or frequencies summary statistics. Normality of distribution will be evaluated using the Kolmogorov-Smirnov test.

Non-normally distributed variables will be transformed into approximately normal distributions by logarithmic transformations and again tested for normality.

The main analysis will be the effect of the intervention on serum ferritin concentrations. Analysis of covariance will be used to compare the kiwifruit and the banana groups at the end of the study while controlling for baseline serum ferritin concentrations. Comparisons will also be made within the kiwifruit and banana groups between baseline and endpoint measures using the dependent t-test. The effect of the intervention on haemoglobin, soluble transferrin receptor, soluble transferrin receptor: serum ferritin ratio, ascorbic acid and carotenoids between and within groups will also be investigated using the statistical methods described above. The data will be investigated for possible confounding factors and effect modifiers e.g. age, body mass index, blood loss and dietary intakes. If confounding factors exist, analysis of covariance will be used to analyse the effect of the kiwifruit compared to the banana on iron status while controlling for the effects of these confounding factors. Serum ferritin, soluble transferrin receptor, soluble transferrin receptor: serum ferritin ratio and ascorbic acid will be tested using one-sided tests. Two sided tests will be used for all other variables. Effect size (to check for type 1 errors) will be calculated and where a statistically non-significant effect is seen, post hoc power calculations will be conducted to find out if the sample size was sufficiently large enough to detect an effect (to check for type 2 errors). Significance will be set at p < .05.

The percentage of days each subject consumed the breakfast will be calculated from compliance diaries. Descriptive statistics will be used to describe subject's evaluation regarding breakfast cereal, milk and fruit consumption.

### Funding and ethics

Funding for the study was provided by ZESPRI^® ^International Ltd. The initial screening of subjects was funded through the Massey University Research Fund and the New Horizons for Women Trust Research Award. Subjects were provided with $50 worth of petrol vouchers for completing the entire study.

Ethical approval was obtained from the Massey University Human Ethics Committee: (Southern A), Reference No.08/20. All subjects signed an informed consent form before participating in this study.

### Provision of results to subjects

On completion of the study, subjects will be informed of their current and baseline iron status. All subjects will be sent a brief report summarising the main findings of the project via email.

## Discussion

It is well known that non haem iron absorption is enhanced by the addition of ascorbic acid to meals [10,11]. Lutein and zeaxanthin have recently been shown to increase iron absorption when added to meals [12]. It is less clear whether this increase in iron absorption is reflected in increased iron stores. Previous studies investigating the effect of ascorbic acid consumed with meals have had several limitations. These include small sample sizes, interventions of short duration, and the use of subjects with normal rather than low iron stores. Few of these studies have reported the iron content of the meals to which ascorbic acid was added or added ascorbic acid to meals containing appreciable amounts of iron. Ascorbic acid's mechanistic action for enhancing iron absorption means that ascorbic acid is more likely to improve iron status if added to a meal containing a substantial amount of iron.

The breakfast cereal was chosen as a suitable vehicle to fortify with iron [33], and as a food item that is acceptable to consume with fruit. In New Zealand, sixty percent of women in the most recent National Nutrition Survey aged 25-44 years consumed breakfast cereal at least once per week [2]. It is recognised that the phytic acid content of the breakfast cereal will inhibit iron absorption [34]. An ascorbic acid to iron molar ratio of 4:1 is needed to increase iron absorption from fortified foods high in phytic acid [20]. The ascorbic acid to iron molar ratio of the breakfast meal provided in this study was 3.7:1.

As far as we are aware, this is the first study to investigate the effect of an iron fortified breakfast cereal and kiwifruit on iron status in healthy women with low iron stores and the largest study to investigate the effect of ascorbic acid from food on iron status.

If successful in improving the iron status of these women, the addition of an ascorbic acid rich fruit to an iron fortified breakfast cereal may be an effective method of addressing the issue of iron deficiency in young women.

## List of abbreviations used (if any)

Abs: absorbance; AOAC: association of analytical chemists; BMI: body mass index; CBC: complete blood count; CIHAT: computerised iron habits assessment tool; C-RP: c-reactive protein; EDTA: ethylene diamine tetraacetic acid; Hb: haemoglobin; FACT: food action rating scale; Fe^2+^: ferrous iron; Fe^3+^: ferric iron; FFQ: food frequency questionnaire; HPLC: high performance liquid chromatography; ISAK: international society for the advancement of kinanthropometry; NZPAQ-SF: New Zealand physical activity questionnaire short form; ORAC: oxygen radical absorbance capacity; SD: standard deviation; SF: serum ferritin; SLS-Hb: sodium lauryl sulphate-Hb; SST: Serum Separator Tube; sTfR: soluble transferrin receptor; UV: ultraviolet.

## Competing interests

This research is funded by ZESPRI^® ^International Ltd.

## Authors' contributions

KB was the main author and project leader together with CC. All authors were involved in conceptualising and design of the research project, the acquisition of data and revising the manuscript. All authors read and approved the final manuscript.

## Pre-publication history

The pre-publication history for this paper can be accessed here:

http://www.biomedcentral.com/1471-2458/10/36/prepub
